# Identification and Characterization of Plant-Interacting Targets of Tomato Spotted Wilt Virus Silencing Suppressor

**DOI:** 10.3390/pathogens10010027

**Published:** 2021-01-01

**Authors:** Ying Zhai, Prabu Gnanasekaran, Hanu R. Pappu

**Affiliations:** Department of Plant Pathology, Washington State University, Pullman, WA 99164, USA; ying.zhai@wsu.edu (Y.Z.); prabu.gnanasekaran@wsu.edu (P.G.)

**Keywords:** affinity purification, calmodulin, carbonic anhydrase, heat shock protein, importin, mass spectrometry, NSs, protein–protein interaction network, RNA silencing suppressor, tomato spotted wilt virus

## Abstract

Tomato spotted wilt virus (TSWV; species *Tomato spotted wilt orthotospovirus*) is an economically important plant virus that infects multiple horticultural crops on a global scale. TSWV encodes a non-structural protein NSs that acts as a suppressor of host RNA silencing machinery during infection. Despite extensive structural and functional analyses having been carried out on TSWV NSs, its protein-interacting targets in host plants are still largely unknown. Here, we systemically investigated NSs-interacting proteins in *Nicotiana benthamiana* via affinity purification and mass spectrometry (AP-MS) analysis. Forty-three TSWV NSs-interacting candidates were identified in *N. benthamiana*. Gene Ontology (GO) and protein–protein interaction (PPI) network analyses were carried out on their closest homologs in tobacco (*Nicotiana tabacum*), tomatoes (*Solanum lycopersicum*) and *Arabidopsis* (*Arabidopsis thaliana*). The results showed that NSs preferentially interacts with plant defense-related proteins such as calmodulin (CaM), importin, carbonic anhydrase and two heat shock proteins (HSPs): HSP70 and HSP90. As two major nodes in the PPI network, CaM and importin subunit α were selected for the further verification of their interactions with NSs via yeast two-hybrid (Y2H) screening. Our work suggests that the downstream signaling, transportation and/or metabolic pathways of host-NSs-interacting proteins may play critical roles in NSs-facilitated TSWV infection.

## 1. Introduction

Tomato spotted wilt virus (TSWV; species *Tomato spotted wilt orthotospovirus*) is the best known member in *Orthotospovirus*, which is the only genus with plant-infecting viruses in the family *Tospoviridae* [1]. Belonging to the order *Bunyavirales*, tospoviruses contain segmented RNA genomes with three single-stranded (ss) RNAs packaged in enveloped virus particles [2]. The large (L) RNA is negative sense, while the medium (M) and the small (S) RNAs possess an ambisense genome organization [3]. As a well-studied and economically important plant virus [4], TSWV causes significant yield losses in a wide range of agronomic and horticultural crops such as beans, lettuce, peanuts (groundnuts), peppers, potatoes, tobacco and tomatoes [5,6].

The TSWV L RNA encodes an RNA-dependent RNA polymerase (RdRp). The M RNA encodes a non-structural movement protein NSm, and the precursor of two structural glycoproteins G_N_ and G_C_. A nucleocapsid protein (N) and another non-structural protein (NSs) are encoded by the S RNA [7]. Both the M and S RNAs are organized in an ambisense manner [8]. The three genomic RNAs of TSWV and the N protein form ribonucleoproteins encapsulated by the glycoprotein (G_N_ and G_C_) envelope. TSWV infects plants via the thrips vector in the field [9].

NSs proteins are widely found in plant- and vertebrate-infecting *Bunyaviruses* [10]. NSs proteins from different tospoviruses share a common feature of binding both small and long double-stranded (ds) RNAs [11]. As a non-structural protein, TSWV NSs acts as an RNA silencing suppressor for overcoming the host immunity barrier [12]. NSs is an avirulence determinant of the TSWV resistance gene *Tsw* in peppers [13,14]. *Tsw*-mediated resistance in peppers can be overcome by a single amino acid change in NSs at position 104 (T–A) [15]. The N-terminal domain in NSs is important for its avirulence and RNA silencing suppression functions [16]. Two conserved motifs, GKV/T at positions 181–183 and YL at positions 412–413, are critical for the silencing suppressor function of NSs [7].

Despite the advancement of structural and functional research on TSWV NSs, its protein-interacting targets in host plants are still largely unknown. In this research, we investigated the NSs-interacting proteins in *Nicotiana benthamiana* via affinity purification and mass spectrometry (AP-MS) analysis. Gene Ontology (GO) and protein–protein interaction (PPI) network analyses were carried out on their closest homologs in *Arabidopsis* (*Arabidopsis thaliana*), tobacco (*Nicotiana tabacum*) and tomatoes (*Solanum lycopersicum*). Network analysis was carried out, followed by experimental validation by using the yeast two-hybrid (Y2H) assay. This approach of using AP-MS and network analysis combined with experimental validation offers an efficient approach for understanding the PPIs underlying virus–host interactions.

## 2. Results

### 2.1. Affinity Purification—Mass Spectrometry Analysis Reveals NSs-Interacting Proteins in N. benthamiana

TSWV NSs was fused with an mGFP5 tag at its C-terminal (NSs-GFP) and was transiently expressed in *N. benthamiana* leaves at the four-leaf stage. Its binding proteins were extracted and analyzed by AP-MS. To identify the host proteins that specifically interact with TSWV NSs, overlapping candidates were selected from two independent AP-MS replicates. The list was then compared to the list of candidates that bind the V2 protein of *Croton yellow vein mosaic virus* (unpublished), to remove overlapping non-specific interactors. Eventually, 43 *N. benthamiana* proteins were found to specifically interact with TSWV NSs (Table 1). The list is arranged according to the numbers of peptide spectrum matches (#PSMs), posterior error probability (PEP) values of the PSMs (Sum PEP Score) and sums of the scores of the individual peptides from the Sequest HT search (Score SEQUEST HT) in Replicate 1 (R1).

Multiple signaling-relevant proteins can be found in the NSs-interacting list, including a lectin receptor kinase (LecRK; A0A0K1U1X9), a mitogen-activated protein kinase (MAPK; A0A0C5LA06), a calcium-dependent protein kinase (CDPK; A0A1V1H6S6), a calmodulin (CaM) (U3MW48) and two serine/threonine protein kinases (STPKs; A0A4Y5QRT8 and Q5D1L7). Two heat shock proteins (HSPs), HSP70 (Q769C6) and HSP90 (A0A0M3SBS3), also interact with NSs. For these *N. benthamiana* interactors, their closest homologs in tobacco, tomatoes and *Arabidopsis* were found by BLASTP and are listed in Table 2. Both A0A286RNF7 and A4D0J9 are carbonic anhydrases with LOC107768773, Solyc02g086820 and AT3G01500 being their closest homologs in tobacco, tomatoes and *Arabidopsis*, respectively. Therefore, only 42 inferred homologous proteins in each species are listed (Table 2).

### 2.2. Gene Ontology Overrepresentation/Enrichment Tests of NSs-Interacting Proteins

To facilitate GO analysis, the closest tobacco, tomato and *Arabidopsis* homologs inferred from the *N. benthamiana* NSs-interacting proteins (Table 2) were used for overrepresentation/enrichment tests. Only the *Arabidopsis* homologs generated meaningful results in the GO biological process test that classified proteins according to the cellular activities in which they were involved (Table 3). Defense-responsive proteins were found to be enriched by about 10 fold (Table 3), which is consistent with the virulent nature of NSs. The defense-related proteins in the list include a LecRK (AT5G55830), a carbonic anhydrase (AT3G01500), chloroplast photosystem II subunit P1 (PSBP1; AT1G06680), a CaM (AT3G43810), a lipoxygenase (AT1G55020), STPK PBS1 (AT5G13160), a MAPK (AT4G01370), a cysteine proteinase (AT4G39090), a defensin-like protein (AT1G61070), a calcium-transporting ATPase (AT3G57330) and a NB-LRR protein required for hypersensitive response (HR)-associated cell death (NRC) (AT1G53350). The host immunity responses triggered by these defense proteins may be suppressed by the binding of NSs during TSWV infection.

Meaningful results were obtained when using tobacco and *Arabidopsis* homologs in the overrepresentation test of GO molecular functions (Table 4). In tobacco homologs, proteins that bind unfolded proteins were found to be enriched by more than 20 fold (Table 4), including the nascent polypeptide-associated complex subunit α (LOC107791866), two HSPs (LOC107768797 and LOC107803414) and a DnaJ protein homolog (LOC107801992). NSs may interact with them to prevent the correct folding of host proteins. In *Arabidopsis* homologs, cysteine-type proteinases (also called proteases or endopeptidases) were found to be enriched by about 30 fold (Table 4), including an aleurain-type cysteine proteinase (AT5G60360), a type-II metacaspase (AT1G79330), a KDEL-tailed cysteine endopeptidase (AT3G48340) and a glycinain-type cysteine proteinase (AT4G39090).

Meaningful results were obtained when using tomato and *Arabidopsis* homologs in the GO cellular component overrepresentation test (Table 4). In tomato homologs, lysosomal enzymes localized in the extracellular space were enriched by about 40 to 50 fold (Table 5), which include three cysteine proteases: Solyc07g041900, Solyc02g077040 and Solyc04g080960. Similarly, in *Arabidopsis* homologs, lysosome- and chloroplast-localized proteins were found to be enriched by more than 40 and 7 fold, respectively (Table 5). The three lysosome-localized *Arabidopsis* homologs include an aleurain-type cysteine proteinase (AT5G60360), a KDEL-tailed cysteine endopeptidase (AT3G48340) and a glycinain-type cysteine proteinase (AT4G39090). Actually, all four *Arabidopsis* cysteine proteinases characterized in the GO molecular function test are localized in the lysosome, except the type-II metacaspase (AT1G79330). The chloroplast-localized *Arabidopsis* proteins in the list include a carbonic anhydrase (AT3G01500), PSBP1 (AT1G06680), a glutamate-tRNA ligase (AT5G64050), chloroplast photosystem II subunit O2 (PSBO2; AT3G50820), a glutathione S-transferase (GST; AT1G78380), HSP90 (AT5G56000), a plastid RNA-binding protein (AT3G48500) and glucose-6-phosphate 1-dehydrogenase (G6PD1; AT5G35790).

### 2.3. The Protein-Protein Interaction Network of NSs-Interacting Proteins

**Figure 1:**
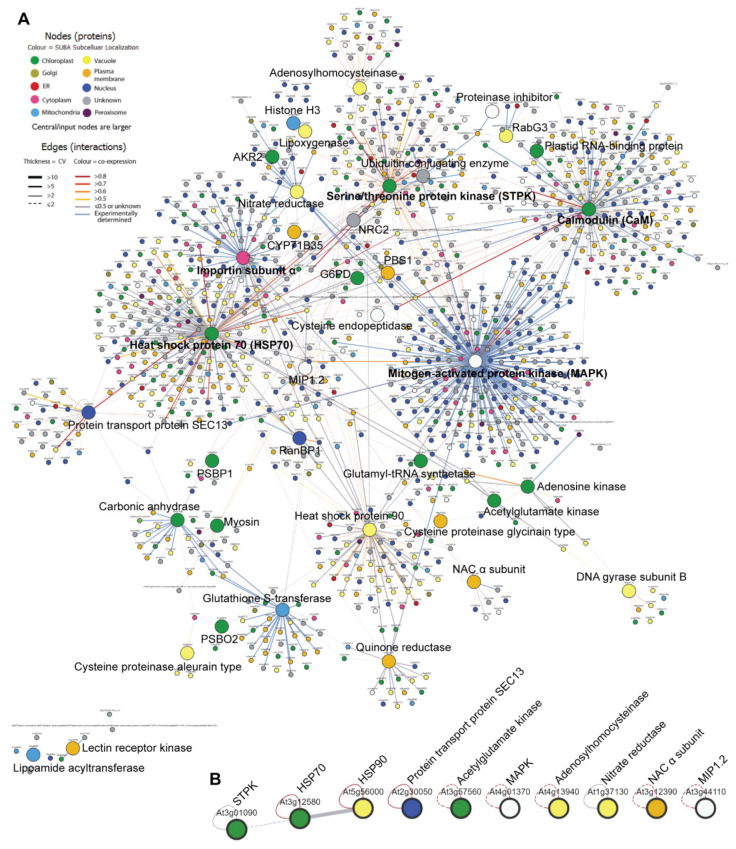
(**A**) The protein–protein interaction (PPI) network of 42 *Arabidopsis* homologs inferred from *N. benthamiana* NSs-interacting proteins. A total of 1346 interactions were predicted. HSP70, CaM, MAPK, STPK and importin subunit α are five major nodes found in the PPI network. (**B**) Predicted interactions within the 42 *Arabidopsis* homologs. The most reliable interaction occurs between HSP70 and HSP90. Ten proteins including HSP70 and HSP90 have self-interactions.

To explore the indirect and expanded consequences of physical interactions between NSs and plant proteins, a PPI network was constructed for 42 *Arabidopsis* homologs inferred from *N. benthamiana* NSs-interacting proteins (Figure 1A and Figure S1; Table S1). A total of 1346 interactions were predicted. Five major node proteins can be found in the PPI network, including HSP70 (At3G12580), CaM (AT3G43810), MAPK (AT4G01370), STPK (AT3G01090) and importin subunit α (AT4G16143) (Figure 1A). Interactions between NSs and these five plant signaling, chaperone and transporter proteins may play significant roles in TSWV infection. We further investigated interactions within the 42 *Arabidopsis* homologs (Figure 1B). The most reliable interaction was predicted to occur between HSP70 and HSP90 (Figure 1B). Ten proteins including HSP70 and HSP90 were predicted to have self-interactions (Figure 1B). As two major nodes in the PPI network, CaM and importin subunit α were selected for the further verification of their interactions with TSWV NSs.

### 2.4. Importin Subunit α and Calmodulin 3 Interact with NSs in Targeted Yeast Two-Hybrid Assays

**Figure 2:**
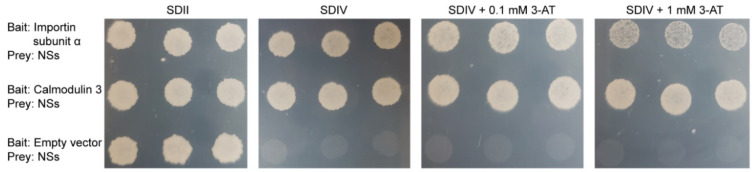
*N. benthamiana* proteins importin subunit α and Calmodulin 3 (CaM 3) were verified to interact with tomato spotted wilt virus NSs via targeted yeast two-hybrid (Y2H). Positive interactions were implied by the yeast’s ability to grow on synthetic defined (SD) selection medium minus four elements of uracil, histidine, leucine and tryptophan (SDIV) and its tolerance to the His3p enzyme inhibitor 3-aminotriazole (3-AT). All yeast clones grow normally on the SDII medium which only lacks leucine and tryptophan. Two concentrations of 3-AT (0.1 and 1 mM) were used in the test.

*N. benthamiana* importin subunit α (A1YUL9) and CaM 3 (U3MW48) were selected to verify their interactions with NSs via targeted Y2H. Both proteins interacted with NSs in the assays (Figure 2), which demonstrates that the AP-MS approach is effective and reliable in identifying host-NSs-interacting proteins.

## 3. Discussion

Although NSs is well-known for its RNA silencing suppressor function during the TSWV infection process, the direct protein-interacting targets of NSs in plant hosts are still largely unknown. There is a report that TSWV NSs can suppress jasmonate signaling in plants [17] via direct interactions with three basic-helix-loop-helix (bHLH) transcription factors (TFs): MYC2, MYC3 and MYC4 [18]. In this work, we significantly expanded the reservoir of NSs’ physical interactors in plants. The interactions may be critical for TSWV virulence.

Multiple NSs-interacting proteins identified in this research have been demonstrated to regulate plant defenses. For example, cysteine proteinases play prominent roles in plant–pathogen interactions [19]. Notably, tomato aleurain-type cysteine proteinase can be inhibited by the pathogenic oomycete *Phytophthora* [20]. NSs-interacting cysteine proteinases are critical for lysosome-mediated autophagy function, which acts as an antiviral defense mechanism in plants. Viruses counteract host defenses by hijacking the autophagy pathway [21]. Interactions between NSs and lysosome-localized cysteine proteinases may contribute to the TSWV-induced suppression of autophagy.

*N. benthamiana* CaM 3 and importin subunit α are two NSs interactors verified by both AP-MS and Y2H assays. CaMs are significant components in plant immunity signaling networks [22]. There are multiple lines of evidence showing that CaMs participate in plant defenses against bacterial [23], fungal [24] and viral [25,26,27,28] pathogens. A tobacco CaM can bind the RNA silencing suppressor encoded by *Cucumber mosaic virus* and thereby trigger its degradation via the autophagy pathway [25]. On the contrary, an *N. benthamiana* CaM is required for the RNA silencing suppressor function of βC1, which is encoded by the geminivirus *Tomato yellow leaf curl China virus* [26]. Thus, the interaction between *N. benthamiana* CaM 3 and TSWV NSs may lead to either NSs degradation or the activation of its RNA silencing suppressor activity. Further investigations would reveal whether CaM 3 plays a positive or negative role in the NSs-mediated suppression of plant RNA silencing.

Importins are critical for the nuclear import of Agrobacterium virulence proteins [29]. There are multiple reports demonstrating that plant importin subunit α facilitates the nuclear transportation of viral proteins. *N. plumbaginifolia* importin subunit α can bind the coat/capsid proteins (CPs) of *Rice tungro bacilliform virus* and *Mungbean yellow mosaic virus* and transport them into the nucleus [30]. Similarly, tobacco importin subunit α mediates the nuclear import of *Cauliflower mosaic virus* translational transactivator protein P6, which suppresses plant RNA silencing in the nucleus [31]. *N. benthamiana* importin subunit α has a similar function of transporting viral proteins. For example, it interacts with the CP of *Beet black scorch virus* and transports it into the nucleus [32]. The nuclear localization of the *Potato mop-top virus* Triple Gene Block1 (TGB1) protein is mediated by *N. benthamiana* importin subunit α, which facilitates systemic virus movement [33]. The *Pelargonium line pattern virus* p37 protein acts as an RNA silencing suppressor whose nuclear localization is also mediated by *N. benthamiana* importin subunit α [34]. Taken together, we postulate that the physical interaction between TSWV NSs and importin subunit α may facilitate the nuclear transportation of NSs and the following exertion of its RNA silencing suppressor activity.

Since many plant virus infection events occur in the chloroplast [35] and are regulated by host photosynthetic and photomorphogenic activities [36], it is not surprising that NSs interacts with multiple chloroplast-localized proteins. Chloroplast-localized carbonic anhydrases appeared twice in our refined *N. benthamiana* NSs interactor list (Table 1). Their antioxidant activity is involved in plant HR defenses [37]. For example, carbonic anhydrase expression is indispensable for potato resistance to the late blight pathogen *Phytophthora infestans* [38]. It is possible that NSs interacts with plant carbonic anhydrases to suppress their antioxidant function, thereby promoting TSWV infection.

Both HSP70 and HSP90 were found to interact with TSWV NSs in our AP-MS analysis (Table 1). HSP70 is a major node in the PPI network of NSs-interacting proteins (Figure 1A). Functional and physical interactions between HSP70 and HSP90 exist ubiquitously in bacteria, yeasts [39] and plants [40]. In *Arabidopsis*, HSP70 expression can be induced by infections by multiple virus species [41]. In tomatoes, the *Tomato yellow leaf curl virus* CP interacts with HSP70 to facilitate virus infection [42]. In *N. benthamiana*, HSP90 is indispensable for plant resistance against *Potato virus X* and *Tobacco mosaic virus* [43]. Based on these reports, HSP70 and HSP90 may interact with NSs to positively or negatively regulate TSWV infection.

Overall, the NSs-interacting proteins identified via AP-MS provide multiple clues for dissecting the roles of NSs in TSWV–host interaction. CaM-triggered defense signaling, importin-facilitated protein nuclear transportation, carbonic anhydrase-catalyzed antioxidation and HSP70/HSP90-mediated stress tolerance emerged as principal plant cellular activities in response to NSs invasion. In the future, the molecular mechanisms of how TSWV NSs interacts with these defense-related proteins (e.g., time and spatial patterns); the genetic, biochemical and physiological outcomes of the interactions; the expression changes of downstream genes triggered by the interactions; and the regulatory/regulated proteins up-/downstream of the interaction cascades should be investigated to obtain more details. The obtained results would provide a comprehensive portrait of NSs’ activities in the plant cell.

## 4. Materials and Methods

### 4.1. Plasmids and Gene Cloning

The TSWV NSs coding sequence (CDS) was previously described [8]. The NSs CDS was amplified using the PCR primers 5′-GGGGACAAGTTTGTACAAAAAAGCAGGCTATGTCTTCAAGTGTTTATGAG-3′ and 5′-GGGGACCACTTTGTACAAGAAAGCTGGGTGTTTTGATCCTGAAGCATA-3′. The amplified NSs CDS was cloned into the Gateway Donor vector pDONR 207 (Invitrogen) via a BP reaction (insertion of the att-B-sequence-containing PCR product into the att P recombination sites) and then inserted into the destination expression vector pEarleyGate 103 [44] from pDONR 207 via an LR reaction (insertion of the att-L-sequence-containing DNA into the att R recombination sites). In pEarleyGate 103, NSs was fused with an mGFP5 tag at its C-terminal (NSs-GFP). All the PCR-amplified sequences used in this research were verified by sequencing.

### 4.2. Affinity Purification—Mass Spectrometry Analysis of NSs-Interacting Proteins

NSs-GFP was transiently expressed in *N. benthamiana* leaves by agroinfiltration. Leaf samples were collected two days after infiltration, and the expression of NSs-GFP was verified by Western blotting. AP-MS was carried out using the GFP-Trap beads (Chromotek, Germany) as previously described [45,46]. Briefly, infiltrated leaves were ground into fine powder in liquid nitrogen, mixed with protein extraction buffer (1 mL per 500 mg of tissue) and then thawed on ice. After incubation and centrifugation at 4 °C, the extract supernatant was cleaned by filtration and then mixed with the GFP-Trap beads. After 1 h of incubation at 4 °C, the mixture was subsequently washed with wash buffer 3–5 times. The mass spectrometry (MS) analysis of the immunoprecipitated proteins was performed at the BGI Americas MS Service Center. The MS data were searched against the most updated Uniprot *N. benthamiana* database (2020_05) [47] using SEQUEST HT 2013 [48].

### 4.3. Refinement of the NSs-Interacting Protein Candidate List

Two NSs-GFP AP-MS biological replicates as well as two non-NSs AP-MS control replicates were performed for NSs-GFP. Since the Gateway-compatible pEarleyGate 103 cannot express mGFP5 without gene insertion, a pEarleyGate 103 construct expressing an mGFP5-fused V2 protein from *Croton yellow vein mosaic virus* was used as the non-NSs AP-MS control. Overlapping NSs-interacting protein candidates were identified from the two NSs-GFP AP-MS replicates. Non-specific NSs interactors were then removed from the list, including mGFP5, ubiquitin and proteins that were found to also interact with V2 in the control samples. This step helped to exclude non-specific NSs-interacting proteins that are expressed at high levels in *N. benthamiana*.

### 4.4. Verification of NSs-Interacting Proteins by Targeted Yeast Two-Hybrid Assay

Two *N. benthamiana* proteins in the interacting list, importin subunit α (A1YUL9) and CaM 3 (U3MW48), were selected to verify their interactions with NSs via targeted Y2H. The Y2H procedure has been described previously [49]. In brief, the NSs CDS was cloned into the Gateway-compatible prey vector pACT2-GW (pACT2-GW-NSs, with leucine selection marker) and then used to transform yeast strain A. After testing the transformed yeast clones for self-activation, the *importin subunit α* or *CaM 3* CDS was cloned into the Gateway-compatible bait vector pBTM116-D9 with tryptophan selection marker and then used to transform a selected yeast line harboring pACT2-GW-NSs. Empty pBTM116-D9 was used as a negative control. Positive interactions were implied by the observation of the yeast’s growth on synthetic defined (SD) selection medium minus four elements of uracil, histidine, leucine and tryptophan (SDIV) and its tolerance to the His3p enzyme inhibitor 3-aminotriazole (3-AT).

### 4.5. Gene Ontology Analysis of Inferred Tobacco, Tomato and Arabidopsis Homologs

Since there is currently no available ontology data and analysis tool for *N. benthamiana*, the GO analysis of NSs-interacting proteins was performed using their closest homologs in tobacco, tomatoes and *Arabidopsis*. Tobacco is a close relative of *N. benthamiana*, and the genome of tomatoes has been well-studied compared to other species in the *Solanaceae* family. However, neither tobacco nor tomatoes have annotation information sufficient for a comprehensive GO analysis. Thus, *Arabidopsis* homologs were still used for all the GO enrichment tests for biological processes, molecular functions and cellular components. The test results for tobacco and tomato homologs were included if they contained meaningful information. All the tests were performed using the PANTHER (Version 15.0) GO Term Enrichment tools [50].

### 4.6. Protein-Protein Interaction Network Analysis of Inferred Arabidopsis Homologs

*Arabidopsis* homologs were used for the protein–protein interaction (PPI) network analysis due to the availability of PPI data and analysis tools. The PPI analysis of the *Arabidopsis* homologs inferred from the *N. benthamiana* NSs-interacting proteins was performed using the Bio-Analytic Resource (BAR) *Arabidopsis* Interactions Viewer [51]. The queries included interaction data and predictions from BioGrid (Version 4.1) [52], IntAct (Version 4.2.16) [53] and BAR (Version 20-04) [51]. The results of protein–DNA interactions from the BAR were also included.

### 4.7. Mass Spectrometry Data Deposit

The original AP-MS dataset (RAW files) and results of NSs-interacting candidates in *N. benthamiana* were deposited in the ProteomeXchange Consortium via the PRoteomics IDEntifications (PRIDE) [54] partner repository, with the dataset identifier PXD022401.

## Figures and Tables

**Table 1:** *N. benthamiana* protein-interacting candidates for tomato spotted wilt virus (TSWV) NSs revealed by affinity purification and mass spectrometry (AP-MS). Two independent replicates (designated as R1 and R2) were performed. The list is arranged according to the values of the numbers of peptide spectrum matches (#PSMs), posterior error probability (PEP) values of the PSMs (Sum PEP Scores), and sums of the scores of the individual peptides from the Sequest HT search (Scores SEQUEST HT) in Replicate 1 (R1). Two bold candidates (importin subunit α and Calmodulin 3) were further confirmed to interact with NSs via yeast two-hybrid assays.

Accession	Description	#PSMs		Sum PEP Scores		Scores SEQUEST HT	
		R1	R2	R1	R2	R1	R2
A0A286RNF7	Carbonic anhydrase	33	28	65.105	67.837	86.19	71.41
A0A0M3SBS3	Heat shock protein 90-3	28	26	50.052	58.461	63.66	48.87
A4D0J9	Carbonic anhydrase (fragment)	19	22	49.892	67.834	53.63	68.49
I3QHX5	Adenosylhomocysteinase	15	9	23.165	17.085	27.23	11.48
I0B7J2	Chloroplast photosystem II subunit O2 (PSBO2)	12	13	25.788	36.984	35.48	25.76
I0B7J5	Chloroplast photosystem II subunit P1 (PSBP1)	10	9	29.376	31.322	28.37	29.27
U5PY93	MP-Interacting Protein (MIP) 1.2	10	6	18.593	13.573	23.1	12.96
Q769C6	Heat shock protein 70 (fragment)	9	4	9.018	7.495	18.44	6.57
U3MY90	Proteinase inhibitor (fragment)	8	10	19.765	31.157	20.94	26
A0A0A7EAV4	Ankyrin repeat containing protein 2 (AKR2)	6	2	10.561	4.414	12.68	2.11
F2Z9R2	Glucose-6-phosphate 1-dehydrogenase (G6PD)	6	2	7.83	2.791	10.65	1.79
A1YUL9	**Importin subunit α**	5	5	13.326	9.718	17.15	6.01
A0A0C4Y3N1	RabG3c protein	5	6	7.097	6.486	8.22	2.17
A0A0S3ANR1	NB-LRR HR-associated cell death (NRC) 2a	5	3	6.318	2.992	1.87	1.63
Q5YLB4	DNA gyrase subunit B	4	1	4.471	0.71	2.38	0
U3MW48	**Calmodulin 3 (fragment)**	3	1	8.901	2.829	10.37	2.46
Q5XPZ0	Adenosine kinase (fragment)	3	3	5.692	5.426	7.01	4.12
R4S2V6	Lipoxygenase (fragment)	3	1	3.467	0.731	2.21	0
A0A387K491	Ran binding protein RanBP1-1b	3	1	2.618	1.051	1.96	0
A0A0K1U1X9	Clade XV lectin receptor kinase	3	8	1.266	1.145	4.89	7.08
F8WQS4	Quinone reductase (fragment)	2	1	4.126	0.807	4.73	0
A0A172WC56	Defensin-like protein 1	2	1	3.97	0.732	2.84	0
A2PYH3	Nascent polypeptide associated complex α	2	3	3.392	4.614	2.27	4.41
Q6XX16	Glutathione S-transferase U2 (fragment)	2	3	2.565	1.677	3.44	0
D6QX33	Plastid RNA-binding protein	2	1	2.326	0.695	0	0
A0A0C5LA06	Mitogen-activated protein kinase	2	2	2.143	1.889	1.65	0
F8WQS2	Acetylglutamate kinase (fragment)	1	2	3.648	2.075	3.27	0
A0A0A7HDA5	Epi-aristolochene dihydroxylase	1	2	3.296	2.77	3.53	1.72
Q18NX4	Nitrate reductase	1	1	2.896	1.947	2.65	0
B0CN62	Myosin VIII-1	1	1	2.364	1.331	2.55	0
W6JJ90	Nuclear pore complex protein Sec13d	1	1	1.971	0.754	2.69	0
Q20KN2	Metacaspase type II (fragment)	1	1	1.969	2.13	0	1.72
Q5D1L7	Serine/threonine protein kinase (fragment)	1	1	1.888	1.185	2.11	0
Q2QFR2	Cysteine proteinase glycinain type (fragment)	1	1	1.627	1.227	2.28	0
C9DFC0	Phytophthora-inhibited protease 1 (fragment)	1	1	1.605	0.698	1.97	1.78
A0A4Y5QRT8	Serine/threonine protein kinase PBS1a	1	2	1.396	1.303	0	1.77
Q2QFR3	Cysteine proteinase aleurain type	1	1	1.191	1.367	0	1.62
A0A024B875	Dihydrolipoamide acetyltransferase component	1	1	1.003	1.596	2.28	0
D5JXY5	Calcium-transporting ATPase	1	1	0.754	0.754	0	0
A0A1V1H6S6	Calcium-dependent protein kinase isoform 2	1	1	0.732	0.801	2.11	0
Q52JJ5	Glutamyl-tRNA synthetase	1	1	0.7	0.789	0	0
A7L4B4	Histone H3	1	2	0.509	1.589	1.76	2.54
V5KY72	Ubiquitin-conjugating enzyme variant	1	1	0.503	0.766	0	0

**Table 2:** The closest homologs of TSWV NSs-interacting candidates in tobacco (*Nicotiana tabacum*), tomatoes (*Solanum lycopersicum*) and *Arabidopsis* (*Arabidopsis thaliana*). Five bold candidates (HSP70, importin subunit α, CaM, MAPK and STPK) are major nodes in the protein–protein interaction (PPI) network.

Description	Closest Homologs in		
	Tobacco	Tomato	*Arabidopsis*
Carbonic anhydrase	LOC107768773	Solyc02g086820	AT3G01500
Heat shock protein 90 (HSP90)	LOC107768797	Solyc12g015880	AT5G56000
Adenosylhomocysteinase	LOC107783029	Solyc09g092380	AT4G13940
Chloroplast photosystem II subunit O2 (PSBO2)	LOC107766588	Solyc02g065400	AT3G50820
Chloroplast photosystem II subunit P1 (PSBP1)	LOC107830202	Solyc07g044860	AT1G06680
MP-interacting protein (MIP) 1.2	LOC107801992	Solyc04g009770	AT3G44110
**Heat shock protein 70 (HSP70)**	LOC107803414	Solyc11g066060	**AT3G12580**
Proteinase inhibitor	LOC107799889	Solyc03g019690	AT1G17860
Ankyrin repeat containing protein 2 (AKR2)	LOC107793888	Solyc01g104170	AT2G17390
Glucose-6-phosphate 1-dehydrogenase (G6PD)	LOC107794892	Solyc07g045540	AT5G35790
**Importin subunit α**	LOC107810574	Solyc01g060470	**AT4G16143**
RabG3 protein	LOC107815360	Solyc03g120750	AT1G52280
NB-LRR HR-associated cell death (NRC) 2	LOC107792680	Solyc10g047320	AT1G53350
DNA gyrase subunit B	LOC107786139	Solyc12g021230	AT5G04130
**Calmodulin (CaM)**	LOC107761764	Solyc10g081170	**AT3G43810**
Adenosine kinase	LOC107790330	Solyc09g007940	AT5G03300
Lipoxygenase	LOC107830099	Solyc01g099160	AT1G55020
Ran binding protein RanBP	LOC107771336	Solyc08g062660	AT5G58590
Lectin receptor kinase	LOC107782584	Solyc03g080060	AT5G55830
Quinone reductase (fragment)	LOC107761412	Solyc10g006650	AT4G27270
Defensin-like protein 1	LOC107831752	Solyc07g006380	AT1G61070
Nascent polypeptide associated complex α	LOC107791866	Solyc10g081030	AT3G12390
Glutathione S-transferase U2	LOC107782951	Solyc07g056490	AT1G78380
Plastid RNA-binding protein	LOC107787150	Solyc03g111050	AT3G48500
**Mitogen-activated protein kinase (MAPK)**	LOC107794128	Solyc01g094960	**AT4G01370**
Acetylglutamate kinase	LOC107803486	Solyc11g005620	AT3G57560
Epi-aristolochene dihydroxylase; CYP71B35	LOC107759261	Solyc04g083140	AT3G26310
Nitrate reductase	LOC107785409	Solyc11g013810	AT1G37130
Myosin	LOC107806983	Solyc02g020910	AT3G19960
Nuclear pore complex protein SEC13	LOC107777830	Solyc02g087300	AT2G30050
Metacaspase type II	LOC107824366	Solyc09g098150	AT1G79330
**Serine/threonine protein kinase (STPK)**	LOC107808522	Solyc02g067030	**AT3G01090**
Cysteine proteinase glycinain type	LOC107760226	Solyc04g080960	AT4G39090
PIP1; cysteine endopeptidase	LOC107774651	Solyc02g077040	AT3G48340
Serine/threonine protein kinase PBS1	LOC107830934	Solyc05g024290	AT5G13160
Cysteine proteinase aleurain type	LOC107784768	Solyc07g041900	AT5G60360
Lipoamide acetyltransferase component	LOC107820956	Solyc01g066520	AT3G06850
Calcium-transporting ATPase	LOC107814306	Solyc04g016260	AT3G57330
Calcium-dependent protein kinase	LOC107805386	Solyc07g064610	AT3G20410
Glutamyl-tRNA synthetase	LOC107774917	Solyc01g112290	AT5G64050
Histone H3	LOC107759185	Solyc01g073970	AT5G65360
Ubiquitin-conjugating enzyme variant	LOC107831808	Solyc04g007960	AT1G70660

**Table 3:** PANTHER overrepresentation test of Gene Ontology (GO) biological processes using *Arabidopsis* homologs inferred from NSs-interacting proteins. A total of 27,416 proteins (GO Ontology database, doi:10.5281/zenodo.3980761) were included in the *Arabidopsis* reference list. Fisher’s exact test with Bonferroni correction for multiple testing was adopted. Only results with Bonferroni-corrected *p* < 0.05 are displayed.

GO Biological Process Complete	*Arabidopsis* Reference #	NSs-Interacting Proteins				
		#	Expected	Fold Enrichment	+/−	*p* Value
Defense response to bacterium	413	7	0.63	11.06	+	9.04 × 10^−3^
Response to bacterium	506	8	0.78	10.32	+	2.75 × 10^−3^
Response to other organisms	1092	12	1.67	7.17	+	1.74 × 10^−4^
Interspecies interaction between organisms	1120	12	1.72	6.99	+	2.28 × 10^−4^
Response to external biotic stimulus	1092	12	1.67	7.17	+	1.74 × 10^−4^
Response to biotic stimulus	1093	12	1.67	7.17	+	1.75 × 10^−4^
Response to stimulus	5567	22	8.53	2.58	+	1.20 × 10^−2^
Response to external stimulus	1509	15	2.31	6.49	+	9.27 × 10^−6^
Defense response to other organisms	805	9	1.23	7.30	+	8.95 × 10^−3^
Defense response	952	10	1.46	6.86	+	4.03 × 10^−3^
Response to stress	3091	18	4.74	3.80	+	6.09 × 10^−4^
Cellular process	11,979	33	18.35	1.80	+	1.67 × 10^−2^
Unclassified	5450	5	8.35	0.60	−	0.00

*Arabidopsis* reference #: number of proteins that are classified in the category out of 27,416 *Arabidopsis* reference proteins. NSs-interacting protein candidate #: number of NSs-interacting proteins that are classified in the category out of 42 candidates; Expected: expected number of NSs-interacting proteins that are classified in the category out of 42 candidates; Fold enrichment: fold enrichment of NSs-interacting proteins that are classified in the category, calculated as #/Expected; +/−: significantly enriched/diluted.

**Table 4:** PANTHER overrepresentation test of GO molecular function using tobacco and *Arabidopsis* homologs inferred from *N. benthamiana* NSs-interacting proteins. A total of 61,238 proteins (GO Ontology database, doi:10.5281/zenodo.4033054) were included in the tobacco reference list. All other test parameters are the same as those in Table 3.

GO Molecular Function Complete	Tobacco Reference #	NSs-Interacting Proteins				
		#	Expected	Fold Enrichment	+/−	*p* Value
Unfolded protein binding	284	4	0.16	24.64	+	4.15 × 10^−2^
Binding	21,517	26	12.30	2.11	+	5.53 × 10^−3^
ATP binding	4591	12	2.62	4.57	+	9.61 × 10^−3^
Adenyl ribonucleotide binding	4708	12	2.69	4.46	+	1.24 × 10^−2^
Adenyl nucleotide binding	4734	12	2.71	4.44	+	1.32 × 10^−2^
Purine nucleotide binding	5258	13	3.01	4.33	+	6.22 × 10^−3^
Nucleotide binding	5870	16	3.35	4.77	+	6.52 × 10^−5^
Small molecule binding	6512	17	3.72	4.57	+	3.66 × 10^−5^
Nucleoside phosphate binding	5870	16	3.35	4.77	+	6.52 × 10^−5^
Purine ribonucleotide binding	5217	13	2.98	4.36	+	5.70 × 10^−3^
Ribonucleotide binding	5285	14	3.02	4.63	+	9.62 × 10^−4^
Carbohydrate derivative binding	5332	14	3.05	4.59	+	1.07 × 10^−3^
Purine ribonucleoside triphosphate binding	5100	13	2.91	4.46	+	4.43 × 10^−3^
Anion binding	6438	16	3.68	4.35	+	2.38 × 10^−4^
Ion binding	11,853	20	6.77	2.95	+	1.59 × 10^−3^
Unclassified	26,668	2	15.24	0.13	−	0.00
**GO Molecular Function Complete**	***Arabidopsis*** **Reference #**	**NSs-Interacting Proteins**				
		**#**	**Expected**	**Fold Enrichment**	**+/−**	***p* Value**
Cysteine-type endopeptidase activity	72	4	0.11	36.26	+	9.91 × 10^−3^
Cysteine-type peptidase activity	102	4	0.16	25.60	+	3.71 × 10^−2^
Catalytic activity	8305	27	12.72	2.12	+	1.06 × 10^−2^
Cation binding	1647	11	2.52	4.36	+	5.00 × 10^−2^
Ion binding	3071	16	4.70	3.40	+	1.04 × 10^−2^
Binding	9721	31	14.89	2.08	+	1.26 × 10^−3^
Protein binding	5109	23	7.83	2.94	+	3.25 × 10^−4^
Unclassified	5502	1	8.43	0.12	−	0.00

Tobacco reference #: number of proteins that are classified in the category out of 61,238 *Nicotiana tabacum* reference proteins. All other column descriptions are the same as those in Table 3.

**Table 5:** PANTHER overrepresentation test of GO cellular components using tomato and *Arabidopsis* homologs inferred from *N. benthamiana* NSs-interacting proteins. A total of 34,637 proteins (GO Ontology database, doi:10.5281/zenodo.4033054) were included in the tomato reference list. All other parameters are the same as those in Table 3.

GO Cellular Component Complete	Tomato Reference #	NSs-Interacting Proteins				
		#	Expected	Fold Enrichment	+/−	*p* Value
Lysosome	49	3	0.06	50.49	+	1.71 × 10^−2^
Lytic vacuole	52	3	0.06	47.58	+	2.02 × 10^−2^
Intracellular membrane-bounded organelle	5532	18	6.71	2.68	+	1.61 × 10^−2^
Membrane-bounded organelle	5782	19	7.01	2.71	+	7.25 × 10^−3^
Organelle	6262	19	7.59	2.50	+	2.30 × 10^−2^
Cellular anatomical entity	9174	26	11.12	2.34	+	7.57 × 10^−4^
Intracellular organelle	6130	19	7.43	2.56	+	1.70 × 10^−2^
Intracellular	7723	25	9.36	2.67	+	1.09 × 10^−4^
Cytoplasm	5053	21	6.13	3.43	+	3.26 × 10^−5^
Extracellular space	62	3	0.08	39.90	+	3.34 × 10^−2^
Unclassified	25,226	16	30.59	0.52	−	0.00
**GO Cellular Component Complete**	***Arabidopsis*** **Reference #**	**NSs-Interacting Proteins**				
		**#**	**Expected**	**Fold Enrichment**	**+/−**	***p* Value**
Lysosome	46	3	0.07	42.57	+	3.98 × 10^−2^
Vacuole	1084	10	1.66	6.02	+	3.03 × 10^−3^
Cytoplasm	14,776	38	22.64	1.68	+	3.25 × 10^−4^
Chloroplast stroma	718	8	1.10	7.27	+	8.34 × 10^−3^
Plastid stroma	730	8	1.12	7.15	+	9.39 × 10^−3^
Whole membrane	830	8	1.27	6.29	+	2.33 × 10^−2^
Membrane	5495	22	8.42	2.61	+	2.28 × 10^−3^
Bounding membrane of organelle	921	8	1.41	5.67	+	4.82 × 10^−2^
Cytosol	3242	22	4.97	4.43	+	1.35 × 10^−7^
Plasma membrane	3529	18	5.41	3.33	+	1.05 × 10^−3^
Cell periphery	4001	19	6.13	3.10	+	1.35 × 10^−3^
Unclassified	1877	1	2.88	0.35	−	0.00

Tomato reference #: number of proteins that are classified in the category out of 34,637 *Solanum lycopersicum* reference proteins. All other column descriptions are the same as those in Table 3.

## Supplementary Materials

The following are available online at https://www.mdpi.com/2076-0817/10/1/27/s1. Figure S1: Protein–protein interaction (PPI) map of 42 *Arabidopsis* homologs inferred from *N. benthamiana* NSs-interacting proteins (the original map of Figure 2A). Table S1: Protein–protein interaction (PPI) network of 42 *Arabidopsis* homologs inferred from *N. benthamiana* NSs-interacting proteins.
