# Supplementary figures and images for: Identification and Characterization of Plant-Interacting Targets of Tomato Spotted Wilt Virus Silencing Suppressor
Source: Pathogens. 2021 Jan 1;10(1):27. doi: 10.3390/pathogens10010027 (PMC7823891; doi:10.3390/pathogens10010027)

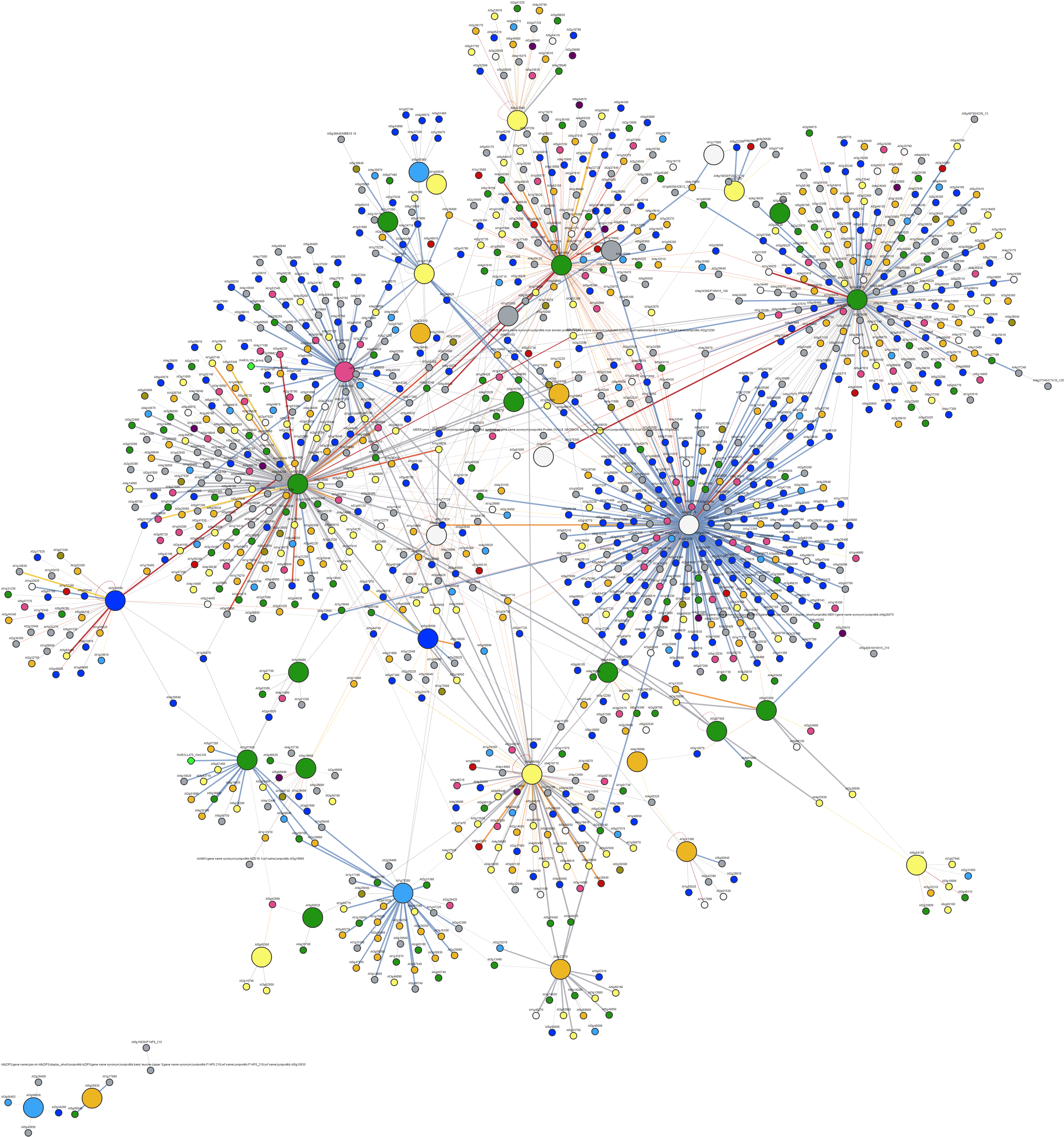

Supplement: Supplementary file 1 [file pathogens-10-00027-s001.zip › Figure S1.png]
